# On residents’ satisfaction with community health services after health care system reform in Shanghai, China, 2011

**DOI:** 10.1186/1471-2458-12-S1-S9

**Published:** 2012-06-22

**Authors:** Zhijian Li, Jiale Hou, Lin Lu, Shenglan Tang, Jin Ma

**Affiliations:** 1 Antai College of Economics and Management, Shanghai Jiao Tong University, 200030, Shanghai, China; 2 School of Public Health, Shanghai Jiao Tong University, 200025, Shanghai, China; 3 Duke Global Health Institute, Duke University, NC 27708, Durham, USA

## Abstract

**Background:**

Health care system reform is a major issue in many countries and therefore how to evaluate the effects of changes is incredibly important. This study measured residents’ satisfaction with community health care service in Shanghai, China, and aimed to evaluate the effect of recent health care system reform.

**Methods:**

Face-to-face interviews were performed with a stratified random sample of 2212 residents of the Shanghai residents using structured questionnaires. In addition, 972 valid responses were retrieved from internet contact. Controlling for sex, age, income and education, the study used logistic regression modeling to analyze factors associated with satisfaction and to explain the factors that affect the residents’ satisfaction.

**Results:**

Comparing current attitudes with those held at the initial implementation of the reform in this investigation, four dimensions of health care were analyzed: 1) the health insurance system; 2) essential drugs; 3) basic clinical services; and 4) public health services. Satisfaction across all dimensions improved since the reform was initiated, but differences of satisfaction level were found among most dimensions and groups. Residents currently expressed greater satisfaction with clinical service (average score=3.79, with 5 being most satisfied) and the public health/preventive services (average score=3.62); but less satisfied with the provision of essential drugs (average score=3.20) and health insurance schemes (average score=3.23). The disadvantaged groups (the elderly, the retired, those with only an elementary education, those with lower incomes) had overall poorer satisfaction levels on these four aspects of health care (P<0.01). 25.39% of the respondents thought that their financial burden had increased and 38.49% thought that drugs had become more expensive.

**Conclusion:**

The respondents showed more satisfaction with the clinical services (average score=3.79) and public health services/interventions (average score=3.79); and less satisfaction with the health insurance system (average score=3.23) and the essential drug system (average score=3.20). Disadvantaged groups showed lower satisfaction levels overall relative to non-disadvantaged groups.

## Background

### Policy background

After the economic reform, which was launched in late 1970s, China's health care system has experienced several significant changes that have had profound implications on access to health care for the population [1]. From the early 1980s to the early 2000s, the financing of health care has gradually been driven by the introduction of a market economy, which had replaced a planned economy. Such a change in the financing of health care system has been seen as unsuccessful because since the 1980s and 90s equity in access to health care has worsened and cost escalation has become uncontrollable [1].

Over the past decade, the government of China has recognized these and other challenges in reforming its health care system. In 2009, it initiated a new round of health care system reforms which aimed to ensure that the State plays a critical role in guaranteeing universal coverage of essential health care and providing secure, efficient, convenient and affordable basic health care services [2]. Four aspects of health care system covering both urban and rural residents have been or continue to be either strengthened or established, as follows: 1) health insurance schemes; 2) national essential drug systems; 3) clinical service system; and 4) the public health/preventive service system. There has been significant progress made in these areas over the past three years and the context of each system will subsequently be explained.

First, regarding health insurance schemes, the basic health insurance system has almost attained universal coverage. After the reform, China developed three mainstream schemes: the Urban Employee Basic Medical Insurance (UEBMI), the Urban Resident Basic Medical Insurance (URBMI) and the New Rural Cooperative Medical Scheme (NRCMS). Additionally, Medical Financial Assistance (MFA) was created to assist the poor.

UEBMI, is jointly funded by employers and employees and uses social health insurance mechanisms. It forms risk-pooling units, which are created independently for each city or county/district. URBMI is financed by central and local governments in addition to individual premiums. It began as a pilot program in 2007 and the funding amount per capita is approximately RMB 150 to RMB 500 ($1 US=6.5 RMB) [3]. Like URBMI, NRCMS is jointly funded by central and local governments and premiums; however, it covers rural residents and was started in 2003. A majority of URBMI and NRCMS benefit packages cover only inpatient care, while an increasing number of Chinese cities and counties have expanded their benefit packages to include outpatient care. MFA is a government program providing supplemental support to the poor by paying health premiums and covering extra costs in seeking care for catastrophic illnesses.

Second, the national essential drug policy has been implemented nationwide. The national essential drugs list for primary health organizations comprises 307 medicines including chemical medicines, biological products and proprietary Chinese medicines [4]. The list specifies that primary care facilities in China are not allowed to earn any mark-up on the sale of essential drugs. The policy has been successful in most places of China, as the prices of essential drugs were reduced by approximately 16%-30% since it was implemented [4].

Third, public hospitals, particularly at local level, (i.e. country/district and township/sub-district level) were reformed to improve the efficiency and effectiveness of service provision. In 2009, the Chinese government promised to strengthen more than 300 county-level hospitals (including traditional Chinese medicine hospitals), over 1,000 central township hospitals and more than 12,000 village clinics. The goal is to have at least one general hospital from each county reaching an expected quality of care, and to have one to three standardized key health centers in each township [3].

Fourth, the reform has called for greater equality in the provision of public health interventions/services between urban and rural areas and between rich and poor areas within the next five years. Starting from 2009, actions have been taken to increase regular health checkups for senior citizens over 65; regular growth checkups on infants and children under three years of age; regular prenatal examinations and postnatal visits for pregnant and lying-in women; and guidance for prevention and disease control to patients with diseases such as hypertension, diabetes, mental disorders, HIV/AIDS, and tuberculosis.

Overall, from a supply-side point of view, the reform is generally seen as successful. However, it is also important to determine the extent of the reform’s success as experienced by the demand side. This paper attempts to answer this question by measuring the residents’ satisfaction with community health services. Community health care services are key to the success of the reform because they are closely related to its four dimensions (health insurance system, essential drugs, basic clinical services and public health services). Community health service facilities not only help to maintain community residents’ health by preventing disease, but also provide treatment for frequently-occurring illnesses and chronic diseases, in addition to rehabilitation and other public health services.

### Literature review

There is a substantial amount of literature covering both patient satisfaction [5-12] and patient characteristics; in addition to aspects of care provided by hospitals related to patient satisfaction [13-21]. Bleich and her colleagues [13] used data from 21 European Union countries to investigate the determinants of satisfaction with the health care system as it relates to patient experience. The authors found that satisfaction with the health care system depends more on external factors such as patient expectations, health status and type of care required rather than on the experience of care as a patient. A study undertaken by Kroneman and others [14] assessed the extent to which direct access to health care services influences the satisfaction with general practitioner services in 18 European countries. The results showed that direct accessibility appeared to be important for patient satisfaction.

Several studies have also investigated patient satisfaction with services provided for specific diseases [22-28]. For example, regarding psychiatric services, Henderson and his team [22] compared patient satisfaction with community-based and hospital providers of care. They found that patients were more satisfied with the community-based service. Bentur et al. [23] examined the satisfaction with and access to community care of the chronically ill in Israel. The authors found that chronically ill and healthy respondents experienced the same satisfaction with community care services. Several studies [29-31] discussed the questionnaire’s design, validity and reliability as it related to patient satisfaction which found that nursing and doctor categories were most notable, and several studies approached different aspects of patient satisfaction [32,33].

There are very few studies that evaluate the health care system reform with regard to resident satisfaction, but several studies [34-36] have investigated reform impact in a few specific areas. Polluste et al. [34] evaluated primary health care reform in Estonia from patients’ perspectives using interview data. Boika et al. [35] discussed the impact of health reforms on child health services in Europe. Hunter et al. [36] evaluated primary care reform in Ontario from the family physician viewpoint. There is no comprehensive evaluation that includes each dimension of the Chinese health care system reform from a service users' perspective.

On the other hand, with the deepening understanding of modern scientific management methods, hospital supervisors are paying increasing attention to patient suggestions and the doctor-patient consultation experience. This is because they are both important in a keenly competitive health care service market. Internationally, many scholars [6,9,14-19,33] agree that patient satisfaction investigation and consultation experience investigation can be helpful in making sustainable improvements to medical services.

Currently a number of scholars have been exploring disease-based satisfaction indices by which medical service quality can be evaluated through a set of patient satisfaction indicator systems. Items to be evaluated include consultation environment, waiting time, medical staff attitude, medical technique, and therapeutic effect and medical cost among others. After repeated examination for health service delivery programs, both the Patient Satisfaction Questionnaire (Ware, PSQ) and the Quality of Care Monitors (Carey) were chosen as the basis for developing similar questionnaire [37]. Also, anchoring vignettes (short descriptions of hypothetical situations) are being used to adjust patient expectation for the purpose of achieving effective comparison among populations [38-40]. Finally, most investigations were done by a third-party using different questionnaires for different types of patient in order to ensure validity and effectiveness of the result. Thus, bias caused by complex interests of different stake-holders could be eliminated.

Although focused more on the clinical outcome, the NHS in England uses four disease-specific patient-reported outcome measures (PROMs). PROMs measure quality of care from the patient perspective and calculate the health gain from a certain intervention. PROMs are measures of a patient health status or health-related quality of life and are typically short, self-completed questionnaires providing measurements at a single point in time. The health status information collected from patients before and after an intervention provides an indication of the quality of care delivered. Since PROMs have been made mandatory, the health gain reported from these four interventions has increased (i.e. the quality of care has become better after report was made necessary). Fitzpatrick et al. have outlined seven criteria for PROMS, which are reliability, validity, responsiveness, precision, interpretability, acceptability, and feasibility [41]. However, weaknesses remain in existing satisfaction investigations. Patient satisfaction is always evaluated only through patients’ self-reporting using a questionnaire; however, results are largely influenced by patients’ understanding of the questions, social environment, cultural environment, unpredictable factors during the investigation and individual subjective factors [37]. Patient satisfaction is highly related to patient expectations as patient satisfaction may change, even though the health service is itself unchanged. Patients have different expectations or reference criteria with regard to their health care service [42-45].

## Methods

### Study design

In order to overcome the shortcomings of satisfaction questionnaire design, this study formulated questionnaire and its index system through questionnaire investigation, literature survey and Delphi expert consultation. It was analyzed from qualitative and quantitative view. Items in the questionnaire included basic information from respondents, inquiries into satisfaction with the health insurance system, satisfaction with the essential drug system, satisfaction with the community clinical service, satisfaction with the basic public health service, residents’ perception of the changes in community health care service quality and overall satisfaction with the implementation of health care system reform. One of the goals of this study was to identify the dimensions or items with which residents may or may not be satisfied. Thus, the key parts of the questionnaire included:

• seven questions on demographic factors to provide explanatory variables (sex, age, place of residence, job, education, income, illness suffered in the last two weeks);

• five questions on health insurance system covering ratio and range of reimbursement, convenience assessment to the reimbursement system;

• four questions on the essential drug system covering drug list and satisfaction;

• twelve questions on aspects of the evaluation by residents of the community clinical service covering the reasons for choosing a particular clinic, medical factors (quality of medical care, etc.) and non-medical factors (waiting time, reasonable costs, etc.);

• seven questions on the public health service including the personal health file, health education, free examination (children, the elderly and pregnant women), prevention knowledge and guidance with respect to chronic disease;

• five questions on health care service improvement covering medical environment, attitude, communication, therapeutic effect and medicine demand;

• one question on overall satisfaction with care and services;

• and one open-ended question requesting comments or suggestions that the residents wished to express about the community health service.

### Sampling

Through a stratified sampling method, eighteen districts of Shanghai were divided into three groups according to their location and level of economic development. Subsequently, one district was randomly selected from each group. These included one center district (Luwan District), one inner suburban district (Changning District) and one outer suburban district (Qingpu District).

In each sampled district, three community health service centers were randomly sampled, and 250 respondents from each service center were selected to respond to questionnaires. The sample size is determined by: . Here n is sample size, *σ*^2^ is variance, E is sampling error, *Z_α_*_/2_ is confidence level. In the case of 95% confidence level and the sampling error is less than 5%, the sample size was generally at 246, so the survey choose 250 as the sample size. Trained investigators randomly distributed 2250 surveys and received 2212 effective responses. After website and email responses were included, the survey accumulated an additional 972 completed questionnaires. The online survey was also limited in the same three districts, and its proportion was similar with the field research. In total, 3184 responses were collected through field and online survey. As for the survey, a 5-point scale was used to rate the residents’ satisfaction towards different aspects of the health system. “1” represented very dissatisfied, “2” quite dissatisfied, “3” satisfied, “4” quite satisfied, and “5” very satisfied.

### Limitations of study

The study has several limitations. First, the influence of responses expectations and individual subjective factors was not considered when we designed investigation questionnaire and the email respondents were more well off and educated. Second, the selection of the respondents focused on only one city, Shanghai. Thus the results may not be generalizable to the entire country due to differences between cities and urban and rural distinctions. Third, the reform process has started relatively recently and, as a result, more time may be needed for these policies to take full effect.

### Research instruments

Data were analyzed using an SAS computer statistical package, Version 9.1 (SAS Institute Inc, Cary, NC). The respondents were grouped by sex, age, education, place of residence (the capital city, urban or rural populations) and annual income. The Spearman correlation test was used to estimate the association between variables. We conducted a multivariate analysis of logistic regression to determine the independent effects on the level of satisfaction.

## Results

### Characteristics of study population

The respondents were grouped by sex, age, job status, education, income and whether or not they suffered illness in the two weeks prior to being surveyed. 41.55% were men and 41.81% were above 60 years of age. 45.04% were employed, 42.28% were retired and the rest were students or unemployed. The education distribution showed that 40.91% of those interviewed had elementary or less education (nine years or less), 29.91% had secondary education (twelve years) and 29.18% had a post-secondary education (more than fifteen years). To explain the socio-economic group differences, the sample was divided into four income groups according to their annual income: less than US$ 1,500 (22.75%), US$ 1,500-6,000 (55.58%), US$ 6,000-20,000 (18.67%) and more than US$20,000 (3%).

The age of the respondents was significantly correlated with job status ( ρ =0.61, p<0.001) and education level ( ρ =0.45, p<0.001). Significant correlation was also found between education and income level ( ρ =0.43, p<0.001); education and job status ( ρ =0.39, p<0.001); and between job status and income level ( ρ =0.32, p<0.001). Of the respondents, 1279 or 41.69% suffered illness in the previous two weeks to being surveyed.

Our sample represents the population who see a doctor in community health service center.

### Satisfaction with different services and facilities

Residents’ satisfaction measurement was divided into five dimensions (clinical service, public health service, drug delivery system, health insurance system and overall satisfaction). Table 2 shows the items and corresponding score of each dimension.

**Table 1:** Demographic characteristic of respondents in Shanghai, 2011

Characteristic	N	(%)
Sex(missing=88)		
men	1342	43.35
Age group (missing=34)		
≤18	53	1.68
19-29	354	11.24
30-39	347	11.02
40-49	463	14.70
50-59	616	19.56
≥60	1317	41.81
Place of Birth (missing=89)		
Shanghai	2518	81.36
Suburban	318	10.27
Other cities	179	5.78
Rural	80	2.58
Employment status(missing=22)		
Employed	1424	45.04
Unemployed	271	8.57
Retired	1337	42.28
Education (missing=34)		
Elementary or less	1289	40.91
Secondary	942	29.91
Post-secondary or above	919	29.18
Income(missing=34)		
Bottom quarter	714	22.75
Second quarter	1744	55.58
Third quarter	586	18.67
Top quarter	94	3.00
Suffer ill in two weeks(missing =116)		
Yes	1279	41.69
No	1789	58.31

**Table 2:** Main areas covered by the satisfaction scale

Dimension	Item	N	Mean	S.D
Clinical services				
	Waiting time	2203	3.73	0.75
	Medical environment	2193	3.86	0.69
	Facility and equipment	2197	3.70	0.75
	Staff attitude	2199	3.98	0.69
	Communication	2197	3.90	0.70
	Therapy effect	2193	3.81	0.70
	Medical cost	2203	3.65	0.77
Public health services				
	Community health education	2801	3.73	0.80
	Community prevention knowledge	2798	3.74	0.77
	Free examination for the elder	2501	3.70	0.79
	Free examination for the children	2220	3.59	0.76
	Free examination for the pregnant	2192	3.59	0.77
	Regular guidance to chronic ill	2597	3.74	0.79
Essential drug system				
	Accessibility to medicine	1688	3.27	0.68
	Medicine price	2680	3.21	0.94
Health insurance				
	Ratio of reimbursement	1668	3.30	0.80
	Convenience to reimburse	1642	3.29	0.83
Overall satisfaction		2736	3.23	0.86

Note: To measure the satisfaction of different dimensions, a five-point scale was used.

As seen in Table 2, after calculated the average scores of the subareas for each of the four categories, we found that the residents were generally satisfied to a relatively high degree with the clinical service, the next highest level was with the public health service; and the results showed less satisfaction with the essential drug system and the health insurance system. The highest satisfaction was with staff attitude (3.98±0.69), and the next highest was with communication between physician and patient (3.90±0.70) and the medical environment (3.86±0.69). Residents were least satisfied with the drug prices (3.21±0.94), accessibility to drugs (3.27±0.68), convenience of reimbursement (i.e. paid by the insurance or the government) (3.29±0.83) and ratio of reimbursement (3.30±0.80). The overall satisfaction related to the health care system was just above the median score 3 (3.23±0.86).

### Services satisfaction by different residents

Satisfaction scores for different dimensions and groups are shown in Table 3. The individual item satisfaction scores were aggregated into total mean scores for each of the four dimensions. The mean score for each dimension was analyzed by sex, age, place of birth, work status, education and income. Judged by different dimension scores, the results show that residents were less satisfied with the essential drug system and the health insurance system relative to the systems related to clinical and public health services/interventions. Scores were 3.20, 3.23, 3.79 and 3.62 respectively. The unemployed, those earning low incomes, born in a suburban setting and having received an elementary education or less, were found to have relatively low satisfaction with all four dimensions of the health care system. Residents who were women, aged over 60, retired, born in urban settings, and in the third-quarter income level (US$ 6,000-20,000), were more satisfied with the medical services. Residents who were women, aged over 60, retired, born in urban settings, received a secondary education and at the second quarter income level (US$ 1,500-6,000) were more satisfied with the public health services/interventions. Residents, who were men, aged under 60, born in rural settings, possessing an elementary or less education and at the third quarter income level were more satisfied with the essential drug system. Residents, who were under 60 years of age, workers not originally from Shanghai, employed, possessing a secondary education and at the third quarter income level were more satisfied with the health insurance system. The difference in the overall satisfaction score between groups was smaller, as shown in the last column of Table 3. On all scales, the community health care system earned mean scores of 3.20. The score was marginally above the midpoint (a score below 3 would indicate a negative evaluation).

**Table 3:** Comparison of satisfaction between dimensions and groups

	Mean Score of the Community Residents’ Satisfaction with the Health Care System				
	Clinical Service	Public Health Service	Essential drug System	Health insurance	Overall Satisfaction

**Sex**					
Men	3.79	3.65	3.23	3.30	3.25
Women	3.85	3.71	3.20	3.30	3.22
**Age**					
≤60	3.81	3.63	3.23	3.33	3.25
≥60	3.83	3.73	3.19	3.21	3.20
**Place of birth**					
Urban	3.84	3.72	3.23	3.23	3.24
Suburban	3.66	3.51	3.08	3.15	3.18
Migrant worker	3.70	3.53	3.31	3.35	3.23
Rural	3.74	3.52	3.25	3.27	3.27
**Employment**					
Employed	3.74	3.56	3.25	3.38	3.23
Unemployed	3.65	3.37	3.15	2.61	2.89
Retired	3.89	3.84	3.20	3.35	3.27
**Education**					
Elementary or less	3.82	3.39	3.24	3.19	3.23
Secondary	3.82	3.73	3.23	3.34	3.26
Post-secondary or above	3.82	3.63	3.15	3.29	3.16
**Income**					
Bottom quarter	3.68	3.48	3.20	3.10	3.13
Second quarter	3.86	3.75	3.19	3.34	3.24
Third quarter	3.91	3.74	3.33	3.43	3.34
Top quarter	3.76	3.72	3.02	3.20	2.99

Average score	3.79	3.62	3.20	3.23	3.20

Note: To measure the satisfaction of subgroups, a five-point scale was used.

### Factors associated with the residents’ perceptions of service improvement

The study further investigated the residents’ perceptions to the health service improvement. Out of all the respondents, 47.64% believed their access to drugs to have either not changed or decreased. Only one-third believed that it had changed for the better, and 19.81% considered it difficult to estimate. In terms of health insurance coverage, more than 53% of respondents thought that little had changed; more than 8% of respondents believed that the reimbursement rate had lowered; only 15.37% of respondents thought that the rate had increased, and 22.86% thought it was difficult to determine.

Residents’ perception with out-of-pocket expenditures and medicine prices was even lower. The percentage of respondents who thought they changed for the worse in these two aspects were 25.39% and 38.49%, respectively; 37.66% and 27.89% of respondents thought that there were no significant changes; only 17.14% and 14.82% felt that they changed for the better; and 19.81% and 18.80% thought the determination was difficult to make. Thus, compared with the price of medicines and the ratio of reimbursement, people have a relative high assessment on the medical environment, medical level and staff attitude; more than half the interviewers reported positive response. This result is thought to have arisen because the patients pay for the services mostly on a fee-for-service basis, so there was no incentive for the health service provider to control the costs. Although the government took some measures to control medicine and service prices, the result was still unsatisfactory according to the survey results. 60.61% of the retired and 49.49% of the unemployed reported negative response to the essential drug system. Indeed, even for the employed respondents, the degree of perception improvement with the essential drug delivery system was obviously lower than with other items. The details are shown in Table 4.

**Table 4:** Respondents’ perceptions to the change of the health service improvement

	Opinion(percentage)			
	Change worse	No change	Change better	Hard to say
Medical environment	1.34	34.57	55.21	8.88
Technical level	1.15	34.42	53.92	10.52
Staff attitude	1.04	28.22	62.45	8.29
Therapy effect	1.14	34.82	48.65	15.39
Medicine demand	10.64	36.82	32.73	19.81
%of reimbursement	8.17	53.60	15.37	22.86
% of out of pocket payment	25.39	37.66	17.14	19.81
Medicine price	38.49	27.89	14.82	18.80

### Relationship between satisfaction and resident characteristics

The mean score for each dimension was analyzed by sex, age, place of residence, work status, education and income. From the results of the logistic regression models, being male, over age 50, from a rural setting, being retired, having an elementary education and having a low level of income were found to have an independent negative effect on every dimension of the health service system (P<0.01). There were significant positive effects (P<0.01) for local farmers, migrant workers, those with higher education level and high income. In particular, the migrant worker group reported greater satisfaction because they generally have graduated from university and can find stable jobs in cities. The results show that vulnerable groups have less accessibility to community health services, highlighting the need for more attention to be paid to this area during the reform process. The analysis results are shown in Table 5.

**Table 5:** Satisfaction with the four dimensions of the health service system and with the overall evaluation, by background variables (logistic regression)

	Clinical Service		Public Health Service		Medicine Delivery System		Health Insurance		Overall satisfaction	
	B	Odds ratio (95% CI)	B	Odds ratio (95% CI)	B	Odds ratio (95% CI)	B	Odds ratio (95% CI)	B	Odds ratio (95% CI)

Male	-0.03	0.82 (0.70-0.96)	-0.03	0.94 (0.81-1.10)	-0.05	0.91 (0.78-1.06)	-0.11	0.81** (0.69-0.94)	-0.10	0.82 (0.70-0.96)
Age≥50	-1.19	0.96** (0.70-1.30)	-0.19	0.88** (0.65-1.21)	-0.15	0.87** (0.64-1.18)	-0.05	1.03** (0.75-1.40)	-0.07	0.95** (0.70-1.30)
Alien peasants	-0.08	0.97** (0.55-1.72)	-0.08	0.94** (0.53-1.66)	-0.03	1.01** (0.57-1.79)	-0.001	0.91** (0.52-1.60)	-0.02	0.97** (0.55-1.72)
Local peasants	0.32	1.42** (1.10-1.84)	0.33	1.41** (1.10-1.82)	0.30	1.39** (1.09-1.79)	0.15	1.06** (0.83-1.37)	0.02	1.02** (0.79-1.30)
Retired	-0.37	0.93** (0.58-1.48)	0.23	0.66** (0.41-1.07)	0.06	1.22** (0.77-1.95)	-0.03	0.64** (0.40-1.02)	-0.15	0.93** (0.58-1.48)
Alien worker	0.28	1.26** (0.63-2.50)	0.10	0.92** (0.46-1.84)	0.16	1.34** (0.68-2.65)	0.09	0.95** (0.48-1.85)	0.16	1.25** (0.64-2.46)
Elementary education	-0.38	0.69** (0.53-0.92)	-0.38	0.74** (0.56-0.97)	-0.45	0.77** (0.59-1.01)	-0.37	0.59** (0.42-0.83)	-0.38	0.59** (0.42-0.82)
Post-secondary or above	0.22	1.16** (0.86-1.57)	0.34	1.27** (0.95-1.71)	0.38	1.29** (0.96-1.72)	0.24	1.08** (0.81-1.45)	0.28	1.14** (0.85-1.53)
Bottom quarter income	-0.01	1.38** (1.10-1.72)	-0.01	1.43** (1.14-1.78)	-0.24	1.37** (1.10-1.71)	-0.85	1.42** (1.14-1.77)	-0.06	1.38** (1.10-1.72)
Top quarter income	0.40	2.41** (1.48-3.94)	0.39	2.13** (1.31-3.46)	0.34	1.98** (1.23-3.18)	0.47	2.48** (1.54-3.99)	0.35	2.08** (1.28-3.38)

* P<0.05, ** P<0.01

### Results of the open ended question

In order to understand the satisfaction with community health services comprehensively, the questionnaire designed an open ended question. Compared with the beginning of the reform, the biggest difference is the improvement in terms of convenience, medical environment and staff attitude. Most of the questions focused on access to drugs, the reimbursement rate, out of pocket payment rate and medicine price.

## Discussion

The health care system in China has made some progress since the government initiated the reforms. As noted in Table 3, respondents showed relative high satisfaction with the overall community health services (average score=3.20). However, this finding was not inconsistent across all of the four main dimensions of services. The respondents showed more satisfaction with the clinical services (average score=3.79) and public health services/interventions (average score=3.79); and less satisfaction with the essential drug system (average score=3.20) and health insurance system (average score=3.23). Only one-third of respondents found the drug delivery system to have improved, and one-third believed that there was no change. More than half the respondents thought that the health insurance system had undergone no change or worsened.

The residents report lower satisfaction with the essential drug system. Out of the 307 medicines on the list, only 205 of them are chemical and biological medicines (the rest are traditional Chinese medicines). The list is too narrow to meet the residents’ demand for basic medicines. Particularly, demand is unmet for medicines used to treat children and chronic illnesses. It was found that the health insurance system should enhance the reimbursement ratio through lowering the deductible, raising the ceiling amount covered and/or expanding the reimbursement range of diseases, surgery and medical examination in order to enhance the level of protection [46].

The results also demonstrate that disadvantaged groups (the elderly, retired, those only with an elementary level of education and those earning a lower level of income) expressed poorer response in overall satisfaction across every dimension. This result of vulnerable groups reporting less satisfaction seems logical and consistent with the reality of China. As most hospitals mainly received payment based on a fee-for-service basis, they have little incentive to control costs, although an increasing number of hospitals are now paid for by other provider payment methods. Additionally, exacerbating the problem, the health insurance system simply makes payments and does not play intentional role in constraining medical costs (average score=3.21). Finally, the ratio of reimbursement (average score=3.30) did not increase greatly since the reform.

The government chose a tender system in order to decrease the price of medicines [47] and to hopefully make the medicine procurement process more transparent and fair. However, there are still many problems with the implementation of the reforms leading to the policies not fully realizing the desired effect. For example, problems include a single supplier monopoly in one city, collusion between a hospital and pharmaceutical manufacturer and between a supplier and health bureau, and a lack of supervision by the local government [48].

Additionally, the residents generally showed a high degree of satisfaction with communication (average score=3.90) and staff attitudes (average score=3.98). It was found that appropriate levels of communication can reduce patients’ anxiety and increase their satisfaction with health care services. This is consistent with previous studies [31]. Although, residents’ satisfaction with the clinical services and public health services appears to be greater overall than satisfaction with the medicine supply system; there were still some items with regard to the medical care service and public health service which had satisfaction values lower than average (e.g. the equipment and facility, the medical cost, waiting time, free examination to for children and pregnant women).

For this reason, it is recommended that the government should concentrate on improving the service related to the above factors to produce greater satisfaction (e.g. vulnerable groups and the price of medicine). The government should also promote fairness and accessibility of medical service between different groups. Additionally, it needs to determine all possible avenues to increase the income of the poor(less than US$1,500 and US$1,500-6,000), reduce unemployment, decrease medicine prices and increase the ratio of reimbursement. This study also shows that, for the elderly, additional financial subsidization must be supplied by government. Quality of care was another important aspect of residents' perception of care in all the study items, and it was also an important factor affecting the residents’ satisfaction. It is necessary to further improve medical staff education through technical training in order to increase the quality of therapy and thus to meet residents’ demands.

## Conclusion

In general, from a demand-side point of view, the reform has made some progress and residents’ satisfaction has improved since the government initiated the new round reform. But differences of satisfaction level were found among most dimensions and groups. Residents are less satisfied with the provision of drugs at urban community health service centers and health insurance schemes, compared to clinical service delivery systems and public health/preventive services. Disadvantaged groups asserted lower satisfaction levels overall relative to non-disadvantaged groups.

This study provides a practical measure of satisfaction with specific dimensions of health care system. We hope that it will provide health decision-makers with empirical evidence to develop informed policies to tackle these challenges. Further efforts are needed to develop an ever-changing constructive evaluation of patient satisfaction with community health care and with Chinese health care system reform. Thus, these results require further follow-up.

## Authors’ contributions

ZL participated in the design, the acquisition, analysis and interpretation of data, and drafted the manuscript, JH collected the data of the residents and drafted part of the manuscript, JH and LL reviewed the manuscript, ST reviewed the manuscript and provided some comments, as well as participating in the finalization of the paper, JM participated in the design and reviewed the manuscript. All authors have read and approved the final manuscript.

## Competing interests

The authors declare that they have no competing interests.
